# Supplementary material for: The peer effects of resident stock market participation: Evidence from 2019 CHFS
Source: PLoS One. 2024 Apr 10;19(4):e0298894. doi: 10.1371/journal.pone.0298894 (PMC11006169; doi:10.1371/journal.pone.0298894)
Supplement: Attachment — Submitted filename: plose one review 2.docx [file pone.0298894.s001.docx]

Abstract should include an over view of research methodology adopted in research paper.

There is a grammatical mistake in 10^th^ line of abstract i.e. “thed stock”.

Elaborate the introduction section by providing a comprehensive discussion about individual stock market participation. Provide a strong case in this section as to why individual stock market participation is needed. Also provide problem statement and need of carrying out this research study with strong recent justifications from literature. Author old reference citations from literature, the problem statement should be further elaborated by providing most recent citations from literature review.

Provide literature support for hypothesis development for each individual stock market participation factor of the research study. Provide recent and updated reference from literature review.

Provide justification that why only “China Household Finance Survey and Research Center (CHFS) in 2019” has been used as a database. Why some other most recent survey are not taken as a database.

Provide justification of why “three-stage, stratified, proportional-to-population size (PPS) sampling method” is used for data collection.

Operational definitions of variables used in the research study should be provided

There is no discussion of diagnostic statistics used in data analysis section. Author must incorporate various diagnostic statistics methods in order to understand various characteristics of data used for analysis.

Discussion in the data analysis section should be supported with references from literature review and acceptance and rejection of hypothesis should be properly highlighted.

There should be uniformity in the reference style given in references section of the research study.
